# Preclinical identification of acute coronary syndrome without high sensitivity troponin assays using machine learning algorithms

**DOI:** 10.1038/s41598-024-60249-6

**Published:** 2024-04-29

**Authors:** Andreas Goldschmied, Manuel Sigle, Wenke Faller, Diana Heurich, Meinrad Gawaz, Karin Anne Lydia Müller

**Affiliations:** Department of Cardiology and Angiology, University Hospital of the Eberhard Karls University Tuebingen, Otfried-Mueller-Str.10, 72076 Tübingen, Germany

**Keywords:** Preclinical ACS, Machine learning, STEMI, NSTEMI, Acute coronary occlusion, Cardiology, Computer science

## Abstract

Preclinical management of patients with acute chest pain and their identification as candidates for urgent coronary revascularization without the use of high sensitivity troponin essays remains a critical challenge in emergency medicine. We enrolled 2760 patients (average age 70 years, 58.6% male) with chest pain and suspected ACS, who were admitted to the Emergency Department of the University Hospital Tübingen, Germany, between August 2016 and October 2020. Using 26 features, eight Machine learning models (non-deep learning models) were trained with data from the preclinical rescue protocol and compared to the “TropOut” score (a modified version of the “preHEART” score which consists of history, ECG, age and cardiac risk but without troponin analysis) to predict major adverse cardiac event (MACE) and acute coronary artery occlusion (ACAO). In our study population MACE occurred in 823 (29.8%) patients and ACAO occurred in 480 patients (17.4%). Interestingly, we found that all machine learning models outperformed the “TropOut” score. The VC and the LR models showed the highest area under the receiver operating characteristic (AUROC) for predicting MACE (AUROC = 0.78) and the VC showed the highest AUROC for predicting ACAO (AUROC = 0.81). A SHapley Additive exPlanations (SHAP) analyses based on the XGB model showed that presence of ST-elevations in the electrocardiogram (ECG) were the most important features to predict both endpoints.

## Introduction

Chest pain is one of the most common preclinical consultations in emergency medicine^1^. Differential diagnosis ranges from benign musculoskeletal causes to life threatening conditions like acute coronary syndrome (ACS) and acute coronary artery occlusion (ACAO) with immediate need for revascularization. In Germany, a preclinical emergency physician is called to patients with suspected ACS and serves as first care provider in this setting. Depending on the patient’s medical history, recent physical examination and electrocardiogram (ECG) findings, the treating emergency physician decides if the patient requires further treatment and hospital admission. The first care provider also determines the location of the hospital that would provide fast and efficient patient care and the best therapeutic options. However, immediate coronary catheterization is not available in all hospitals, therefore, the choice of hospital admission and location has to be made carefully. European guidelines recommend primary percutaneous coronary intervention (PCI) without delay in patients with ST-elevation myocardial infarction (STEMI) and immediate invasive (< 2 h), early invasive (< 24 h) or selective invasive strategies in patients with non-ST-elevation myocardial infarction (NSTEMI) depending on their clinical risk assessment. The GRACE score (Global Registry of Acute Coronary Events) is recommended to help determine clinical risk in this patient population^2,3^. Cardiac troponin levels are a very important biomarker for the diagnosis of myocardial cell necrosis. Serial measurements of troponin I or troponin T are especially useful in the early diagnosis of patients with ACS to establish the diagnoses and determine the risk for an unfavorable outcome and is also recommended by current guidelines^3,4^.

Risk scores like the “HEART Score” (history, ECG, age, risk factors, initial troponin levels) have been shown to predict the 6-week risk of major adverse cardiac events (MACE)^5^. Sagel et al. ^6^ were able to modify this score and develop the “preHEART” score in order to predict MACE and assist preclinical decision-making using point of care visual troponin immunoassays. However, those rapid troponin immunoassays are not widely available and therefore the HEART and preHEART score have very limited applicability for most emergency medicine providers.

Recently, the use of advanced mathematical algorithms and artificial intelligence in clinical medicine is increasing rapidly and might be especially beneficial for a heterogenous chest pain patient in emergency medicine to correctly identify patients with ACS in need for immediate coronary intervention. Machine learning algorithms have been successfully used in the field of cardiology^7^. They have been shown to accurately predict clinical outcomes in patients who underwent TAVR and predict MACE in young patients with coronary artery disease (CAD)^8,9^.

In emergency medicine, quick decision making by the treating clinician is of utmost importance for optimal patient care and can be a major challenge. As mentioned above, the biomarker troponin is essential in the diagnosis of ACS but usually not available in a preclinical setting. Therefore, machine learning based algorithms could present a cost and time effective way to help guide clinical decisions in an evidence-based way in a setting with limited diagnostic modalities.

In this study, we elucidate the predictive value of several machine learning (ML) models using clinically relevant parameters to verify their correct prediction of MACE and acute coronary artery occlusion (ACAO) and compare them to a modified version of the “preHEART” score comprising classical risk parameters which includes all parameters of the preHEART score without troponin analyses and is referred to as “TropOut” score.

## Methods

### Study population

In this retrospective, monocenter cohort study, treatment protocols by the attending emergency physician were filtered for the diagnosis of “STEMI”, “NSTEMI” and “chest pain” between August 2016 and October 2020. Two locally collaborating rescue stations for emergency medicine admitting their patients to the Emergency Department of the University Hospital Tuebingen, Germany, were included. This yielded 2995 patient cases. After applying the exclusion criteria (age < 18, preclinical death, ambulatory treatment, patient declined transportation, transportation to a medical facility other than the Emergency Department of the University Hospital, Tuebingen, Germany), 2760 cases were included for final analysis. This collective had an average age of 70 years and 58.6% were male.

Preclinical patient data like age, gender, vital signs, patient history, ECG interpretation by the attending emergency physician and administered medication was transferred manually from the treatment protocols to a clinical data base. In-house data like coronary catheter results, diagnosis, vital status at discharge and complications like bleeding or stroke were taken retrospectively from our clinical database. Since our clinic introduced high sensitivity troponin essays during the study period, either troponin or high sensitivity troponin values were taken. Thresholds for pathological troponin I values were as follows: > 37 ng/l for high sensitivity troponin in women, > 57 ng/l for high sensitivity troponin in men and > 0.04 pg/ml for troponin in men and women. Myocardial infarction was defined according to the fourth universal definition of myocardial infarction^3,4,10^. Only patients with Type I myocardial infarction were adjudicated to this endpoint. Adjudication was carried out by reviewing coronary catheter laboratory reports and discharge letters.

The study complies with the declaration of Helsinki and good clinical practice guidelines and was approved by the Ethics Committee at the Medical Faculty of the Eberhard Karls University and at the University Hospital of Tübingen (project number 076/2021B02).

### Study outcome

**Figure 1:**
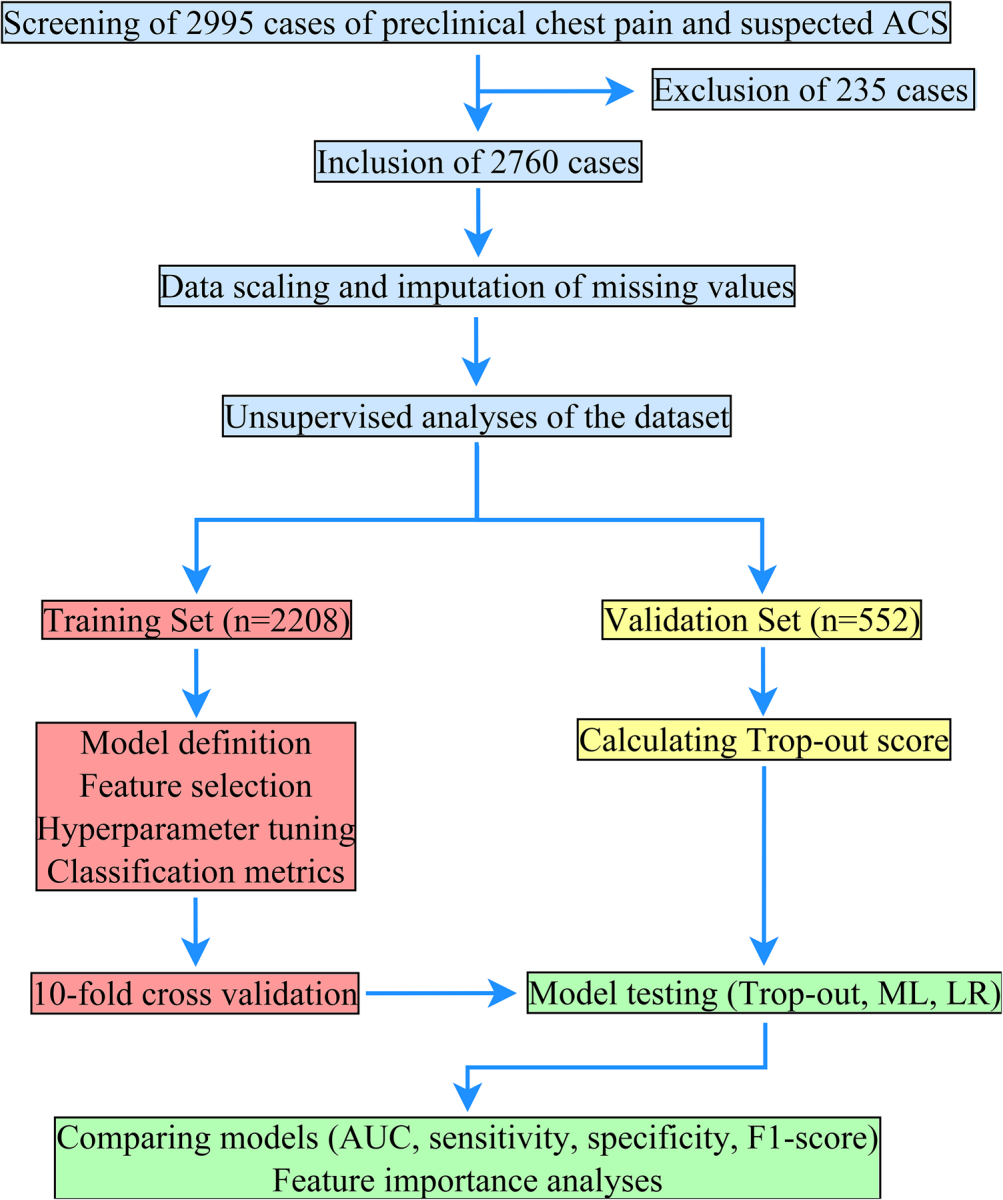
Study flowchart depicting patient recruitment and data processing.

The primary endpoint of this study was prediction of major adverse cardiac events (MACE) at discharge. MACE is a combined endpoint consisting of myocardial infarction, stroke, and death. Our secondary endpoint was acute coronary artery occlusion (ACAO) diagnosed invasively via coronary angiography. Unsupervised analyses and several supervised machine learning models were compared to a modified version of the established preHEART score without the use of cardiac troponin (TropOut score). The preHEART score originally comprises of 5 domains: history (highly: 2 points, moderately: 1 point, slightly suspicious: 0 points), ECG changes (significant ST deviation: 2 points, non-specific repolarisation/LBBB/PM: 1 point, normal: 0 points), age (< 70 years: 2 points, > 40 and < 70 years: 1 point, < 40 years: 0 points), risk (male: 2 points, female: 0 points) and troponin (> 0.05 ng/l: 2 points, 0.03 or 0.04 ng/l: 1 point, < 0.02 ng/l: 0 points). This allows to group patients into high risk for MACE (8–10 points), intermediate risk: 4–7 points and low risk: 0–3 points. Our study design is shown in Fig. 1.

### Statistical analysis

Shapiro–Wilk tests showed that clinical parameters were not-normally distributed. Metric data are reported as median and interquartile range (IQR). Categorical data are given as frequencies and percentages. The Chi square test was used to compare categorical variables whereas Mann Whitney U or Kruskal–Wallis-Test was used to compare metric variables where applicable. Data pre-processing included retrospective calculation of the “TropOut” score. The performance of this score for predicting our primary and secondary endpoint was evaluated by calculating the area und the receiver operating characteristic curve. Classification performance was further evaluated by calculating sensitivity, specificity and F1 score.

Other preprocessing included calculation of body mass index from weight and height and marking input variables as categorical or metric.

Missing values were imputed by mean for continuous data, median for ordinal data and modus for categorical data.

To compare areas under the receiver operating characteristic curve (AUROC) between models, *p*-Values were calculated using the De-Long test**.** Bootstrapping with 1000 random repeats was used to calculate confidence intervals.

Clinical data was analyzed using SPSS version 28.0.0 (https://www.ibm.com/products/spss-statistics). Further statistical analysis was performed in R version 4.0.3 (https://posit.co/) and the package pROC^11^.

### Feature number selection

Parameter reduction was performed for development of our ML models. First, we identified the most relevant features by performing feature importance testing. For this purpose, we both evaluated the impact of the number of selected features on the predictive performance as well as individual feature importance on the model performance, each for the three most common and promising ML models Logistic Regression, Random Forest and XGBoost. Next, the mean importance of scaled importance for the three models was calculated for each variable, and variables were ranked accordingly. Inspecting the mean importance and the plot on performance versus number of selected features yielded the number of features which were used for further analysis.

### Unsupervised data analyses

Unsupervised analyses of the whole cohort were carried out using gower distance as distance metric, in order to calculate similarity and dissimilarity of mixed data types. We then performed dimension reduction on Gower matrices to visualize the whole patient cohort on a two-dimensional space, while preserving individual features of each patient. The outcome variables MACE and ACAO were colored to visualize their distribution over the whole study cohort. Unsupervised machine learning by dimension reduction was performed to gain unbiased insights into all variables of the patient cohort to evaluate if already in the lower dimension projection clusters of patients with similar features are evident. This was not the case for our dataset and underpinned the need to continue with supervised machine learning.

### Machine learning model training and feature importance

Multiple machine learning models were implemented to predicted outcomes using only preclinically available data. Only preclinically acquired variables which included data on age, vital signs, ECG and history were used to train our models. Inner clinical data like laboratory values (for example troponin) were not used to train the models.

Prior to ML model training, feature scaling using z-score normalization was performed on non-categorical data. Following standard ML methodology, the dataset was then randomly split into a training (80% of cases) and validation (20% of cases) dataset. As mentioned above, the primary and secondary outcomes (MACE and ACAO) were used as the output labels.

We investigated the performance of multiple ML classifiers compared to the TropOut score. The following ML models were investigated: random forest with L2 regularization (RF), logistic regression (LR), support vector machine (SVM), gaussian naive bayes (GNB), multi-layer perceptron (MLP), XGBoost (XGB) and AdaBoost (AdaB).

To optimize ML model performance, hyperparameter tuning was carried out using GridSearchCV, a function from scikit-learn^12^.

To optimize performance, we implemented a voting classifier which integrated predictions of all individual models using an ensemble method. We opted for a “soft” voting classifier which combined the predictions of the individual models using equal weight.

To measure and compare performance, we calculated AUC, sensitivity, specificity and F1 score. To plot results, we used receiver operating characteristics (ROC) and precision/recall (PR) curves.

To reduce overfitting, performance evaluation of the training set was carried out using a tenfold cross validation process. To achieve this, training data (80% of the population) was split into 10 groups and the learning process repeated 10 times. The average performance and 95% confidence interval for each ML model is provided in Supplementary Fig. 1.

Once the models were trained, the performance for predicting the primary and secondary outcomes were evaluated on the whole training dataset and finally on unseen data of the validation dataset (20% of the population).

To provide explainability to our model, SHAP values were calculated based on XGBoost. Machine learning models were established with Python 3.10.6 and the packages scikit-learn (1.2.2) numpy (1.22.4), pandas (1.5.3) and matplotlib (3.7.1)^12^.

### Ethical approval

The study complies with the declaration of Helsinki and good clinical practice guidelines and was approved by the Ethics Committee at the Medical Faculty of the Eberhard Karls University and at the University Hospital of Tübingen (project number 076/2021B02). Informed consent was obtained upon hospital admission from all participants.

## Results

### Patients admitted due to chest pain via the emergency department show specific clinical characteristics in association of the underlying disease

We retrospectively studied 2760 consecutive patients admitted to our hospital for chest pain for further diagnostics and treatment. Patients were predominantly male (n = 1616, 58.6%) and the median age was 70 years (IQR 57–80). MACE occurred in 823 (29.8%) patients whereas 480 (17.4%) individuals were invasively diagnosed with ACAO. Patients with MACE and ACAO were also predominantly male when compared to the whole population (*p* < 0.001). Regarding age, individuals with MACE were older whereas patients with ACAO were younger when compared to the whole cohort (*p* = 0.02). Treatment with oral anticoagulation was less common in the ACAO group (*p* < 0.001). Laboratory values TnI, Hs-TnI, CK, CRP and lactate levels were all higher in the ACAO and the MACE group (*p* < 0.001). Unsurprisingly, the MACE and ACAO group showed a higher likelihood of suffering from arterial hypertension and diabetes (*p* < 0.001). The complete baseline characteristics are demonstrated in Table 1.

**Table 1:** Baseline characteristics of patient population.

Parameters	All Patients, N = 2760	MACE, N = 823 (29.8%)	Coronary Occlusion, N = 480 (17.4%)	p
Clinical characteristics				
Age (years)	70 (57–80)	72 (60–81)	68 (57–79)	**0.02**
Gender (male)	1616 (58.6)	555 (67.4)	343 (71.5)	** < 0.001**
BMI (kg/m^2^)	26.8 (30.7–23.8)	26 (24–30)	26.6 (24.1–30.8)	0.864
Cardiac medication on admission				
ß-Blockers	1286 (46.6)	375 (45.6)	213 (44.4)	0.625
ACE-I	863 (31.3)	268 (32.6)	137 (28.5)	0.317
ARB	602 (21,8)	174 (21.1)	98 (20.4)	0.756
Diuretics	724 (26.2)	223 (28.3)	109 (22.7)	0.193
MRA	277 (10)	90 (10.9)	41 (8.5)	0.382
OAC	552 (20)	143 (17.4)	48 (10)	** < 0.001**
ASA	919 (33.3)	266 (32.3)	165 (34.4)	0.742
Platelet aggreg.-I	414 (15)	138 (16.8)	69 (14.4)	0.391
Biomarkers				
Pro BNP (ng/l)	1696 (382–7112)	3062 (720–11,187)	2183 (479–9052)	0.078
TnI (µg/l) (< 0.03 = 0)	0.1 (0.03–0.6)	0.27 (0.60–1.72)	0.44 (0.08–4.66)	** < 0.001**
Hs-TnI (µg/l) (< 3 = 0)	11 (0.03–0.6)	217 (58–1567)	492 (69–3303)	** < 0.001**
CK	104 (69–199)	250 (103–790)	468 (156–1127)	** < 0.001**
CRP (mg/dl)	0.27 (0.06–1)	0.42 (0.11–1.50)	0.44 (0.11–1.41)	** < 0.001**
Lactate	1.5 (1.1–2)	1.8 (1.2–2.6)	1.8 (1.2–2.6)	** < 0.001**
CVRF				
Art. hypertension	2097 (76)	692 (84.1)	398 (82.9)	** < 0.001**
DM	679 (24.6)	255 (31)	140 (29.2)	** < 0.001**
Known CHD	1146 (41.5)	353 (42.9)	206 (42.9)	0.708

Significant values are in bold.

Continuous variables are shown as medians with IQR since they show no normal distribution. Dichotomous variables are presented as frequency with percentages. Kruskal–Wallis tests were used for metric variables whereas chi-square tests were used to compare dichotomous variables.

ACE-I, angiotensin converting enzyme inhibitor; ARB, angiotensin receptor blocker; BMI, body mass index; BNP, b-type natriuretic peptide; CAD, coronary artery disease; CRP, C-reactive protein; MRA, mineralocorticoid receptor antagonist; OAC, oral anticoagulation; Platelet aggreg.-I, Platelet aggregation inhibitor; CK, creatinine kinase; TnI, troponin I on presentation; Hs-TnI, high sensitivity troponin on presentation.

### Preclinically available data was used to develop machine learning models to predict MACE and ACAO

As mentioned in the “Methods” section, optimal number of features was calculated by examining the relationship between number of features included and model output. This way, we included a total number of 26 features. A graph demonstrating this relationship is provided in Supplementary Fig. 2. All features and feature modalities are listed in Supplementary Table 1. These 26 features consisted of preclinical ECG interpretation, age, vital signs and patient history but excluded any inner clinical parameters like laboratory values or echo parameters.

### Unsupervised data analysis reveals heterogenous distribution patterns of clinical risk parameters and insufficient predictive value in patients with chest pain

**Figure 2:**
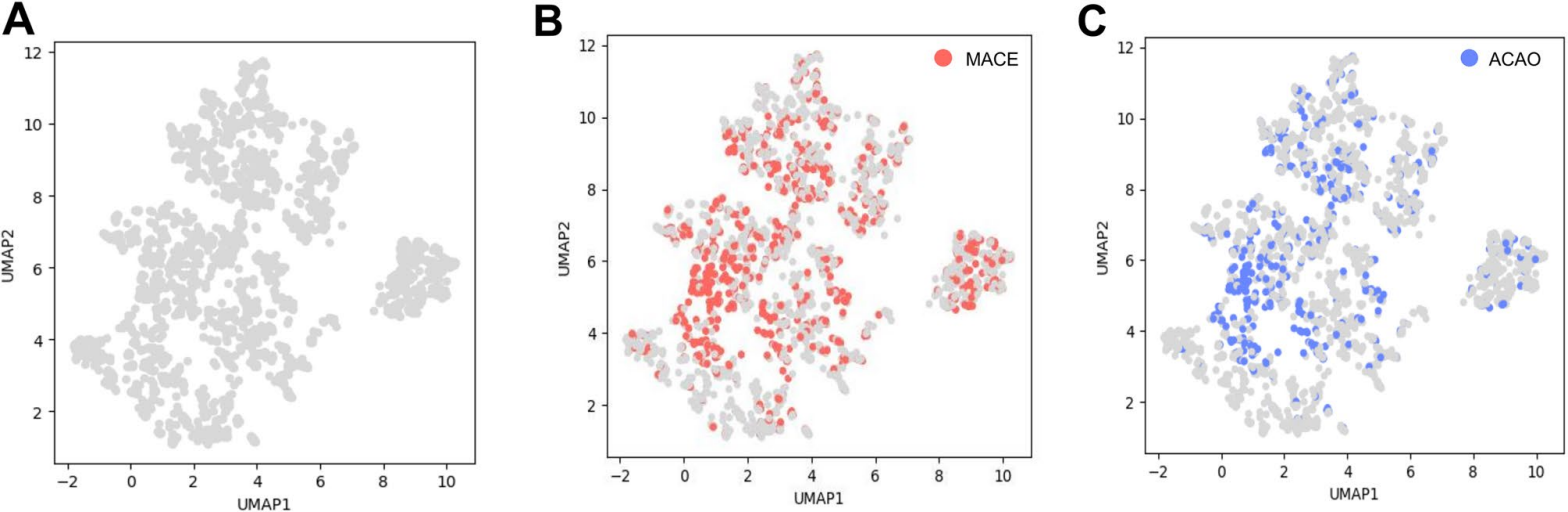
Two-dimensional representation of multidimensional study cohort using Gower distance between patient features and UMAP. (**A**) Dimension reduction by UMAP shows discrete separation into several subclusters. Highlighting patients with MACE (**B**, red) or ACAO (**C**, blue) discloses heterogeneous distribution among patient collective and underpins the requirement of supervised machine learning models to identify patients at risk for MACE or ACAO.

In order to mitigate potential bias and enhance the transparency and understandability of the acquired patient data, we conducted unsupervised machine learning on our entire study cohort prior to analysis. Our aim was to investigate if already the consideration of all patient features and their representation in a two-dimensional space would unveil patient clusters of those being at risk for MACE of ACAO. To this end, all features which were later used for supervised machine learning were subjected to dimension reduction and presented in the two-dimensional space (Fig. 2A). When coloring the outcome variables MACE (Fig. 2B) and ACAO (Fig. 2C), we found a heterogenous distribution of patients at risk, with no specific accumulation in a dedicated cluster. This finding underlines the importance of supervised machine learning, as unsupervised clustering would not be able to decipher patients at risk for MACE or ACAO.

### Supervised machine learning is superior to unsupervised data analysis and serves as independent, reliable prediction tool for the occurrence of a combined cardiovascular endpoint (MACE) in patients with chest pain

After training the ML models on the training dataset (80% of cases, n = 2208), we evaluated their performance to predict MACE on the validation dataset (20% of cases, n = 552). ROC curves including ten-fold cross validation for the ML models for predicting MACE in the training set are shown in Supplementary Fig. 1.

**Figure 3:**
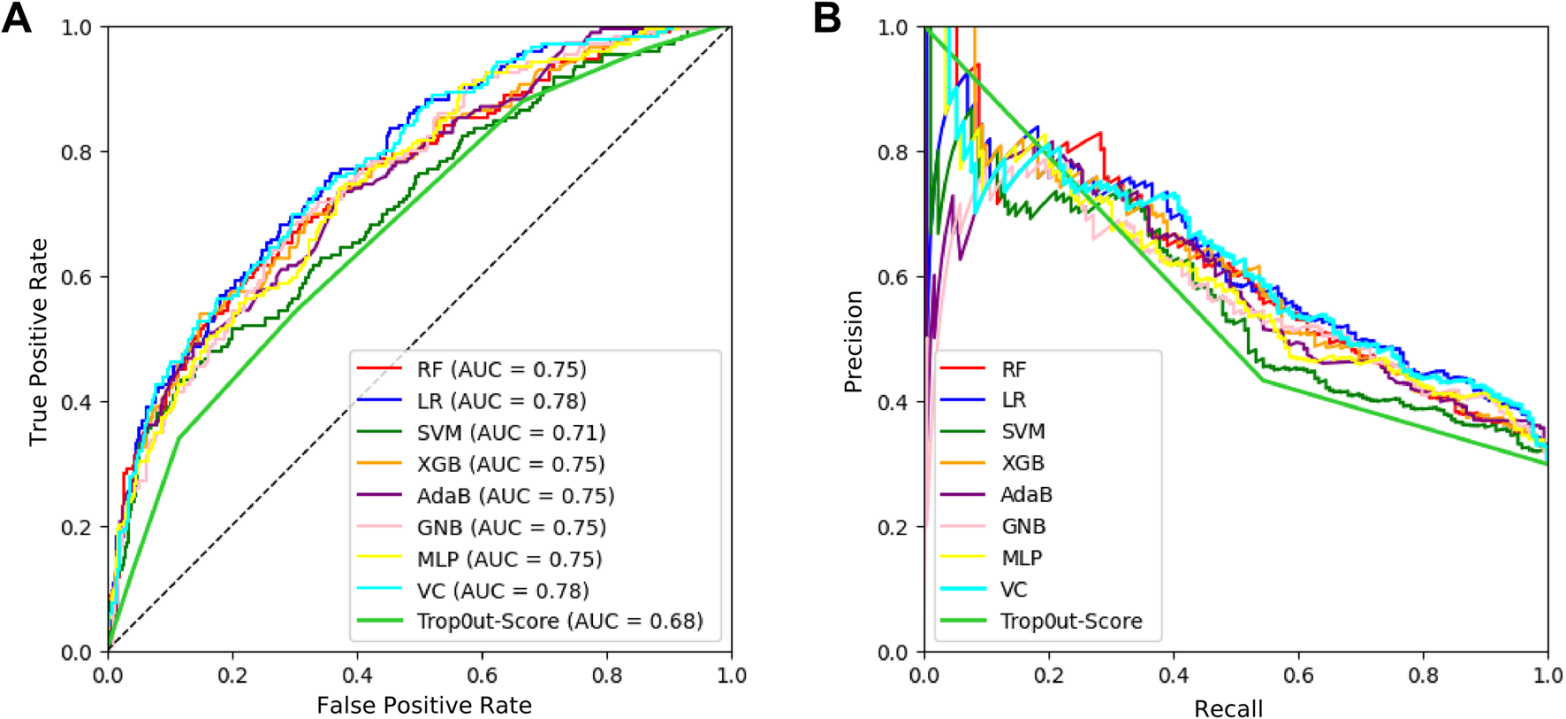
Performance comparison for 7 ML models, a voting classifier integrating all models and the Trop-out-Score for predicting MACE. (**A**) ROC curves visualizing the false positive rate (1-specificity) on the x-axis and the true positive rate (sensitivity) on the y-axis. (**B**) Precision/Recall curves for the same models for predicting MACE. Recall is plotted on the x-axis, precision is plotted on the y-axis. LR, logistic regression; RF, random forest; MLP, multilayer perceptron; GNB, gaussian naive bayes; SVM, support vector machine; VC, voting classifier; XGB, XGBoost; AdaB, AdaBoost.

As mentioned in the “Methods” section, we compared ML models to the Trop-out score (which was also calculated only for the cases of the validation dataset). ROC and Precision/Recall (PR) curves for predicting MACE in the validation set with ML models and the Trop-out score are demonstrated in Fig. 3.

The LR and VC models demonstrated the best discriminative performance with AUROC of 0.78. However, all ML models except for the SVM (AUROC 0.71) were relatively equal in predicting MACE (AUROC ranging from 0.75 to 0.78). In contrast, the Trop-out score showed the worst discriminative performance with AUROC of 0.68.

Additional performance statistics like precision, recall, specificity and F1 score for predicting MACE are demonstrated in Table 2. *p*-Values are calculated by using the De-Long test to compare the AUROC of ML models to the Trop-out score. All ML models except for the SVM showed significantly superior performance when compared to the Trop-out score (*p-values* ranging from < 0.001 to 0.026). We noted high specificity (ranging from 0.91 to 1) for all our ML models except for the GNB (specificity of 0.82).

**Table 2:** Performance of models for predicting MACE.

Model	AUC (95% CI)	Recall (95% CI)	Precision (95% CI)	Specificity (95% CI)	F1-score (95% CI)	*p-Value*
Trop-out	0.68 (0.66–0.69)	0.66 (0.64–0.67)	0.66 (0.64–0.67)	0.70 (0.68–0.73)	0.67 (0.65–0.68)	–
LR	0.78 (0.73–0.82)	0.77 (0.73–0.79)	0.76 (0.72–0.80)	0.95 (0.93–0.96)	0.76 (0.72–0.79)	** < 0.001**
RF	0.75 (0.72–0.78)	0.75 (0.73–0.78)	0.75 (0.73–0.78)	0.98 (0.94–0.98)	0.75 (0.73–0.78)	**0.001**
XGB	0.75 (0.70–0.79)	0.75 (0.70–0.80)	0.75 (0.70–0.79)	0.95 (0.93–0.97)	0.75 (0.70–0.79)	**0.009**
AdaB	0.75 (0.70–0.79)	0.76 (0.734–0.79)	0.76 (0.73–0.79)	0.95 (0.94–0.96)	0.76 (0.73–0.78)	**0.018**
MLP	0.75 (0.70–0.80)	0.75 (0.72–0.77)	0.74 (0.71–0.77)	0.91 (0.89–0.93)	0.75 (0.72–0.77)	**0.026**
GNB	0.75 (0.71–0.80)	0.73 (0.69–0.76)	0.72 (0.68–0.76)	0.82 (0.78–0.85)	0.73 (0.69–0.76)	**0.024**
SVM	0.71 (0.67–0.77)	0.70 (0.65–0.76)	0.49 (0.41–0.57)	1.00 (1.00–1.00)	0.58 (0.51–0.65)	0.762
VC	0.78 (0.74–0.81)	0.76 (0.73–0.79)	0.75 (0.73–0.78)	0.93 (0.91–0.95)	0.76 (0.73–0.78)	** < 0.001**

Significant values are in [bold].

Performance of 8 ML models, as well as the Trop-out score for predicting MACE in the validation dataset (n = 552). Models were compared to the Trop-out score and *p*-values were calculated using the De-Long test. Values in brackets show the 95% confidence intervals and were calculated via a bootstrap method.

LR, logistic regression; RF, random forest; MLP, multilayer perceptron; NB, naive Bayes; SVM, support vector machine; VC, voting classifier; XGB, XGBoost, AdaB, AdaBoost.

### Supervised machine learning serves as reliable predictor for the presence of acute coronary artery occlusion with the need to immediate coronary intervention in patients with chest pain

Similar to MACE, we also evaluated prediction of ACAO by ML models and Trop-out score. ROC curves including ten-fold cross validation for the ML models for predicting ACAO in the training set are shown in Supplementary Fig. 1.

**Figure 4:**
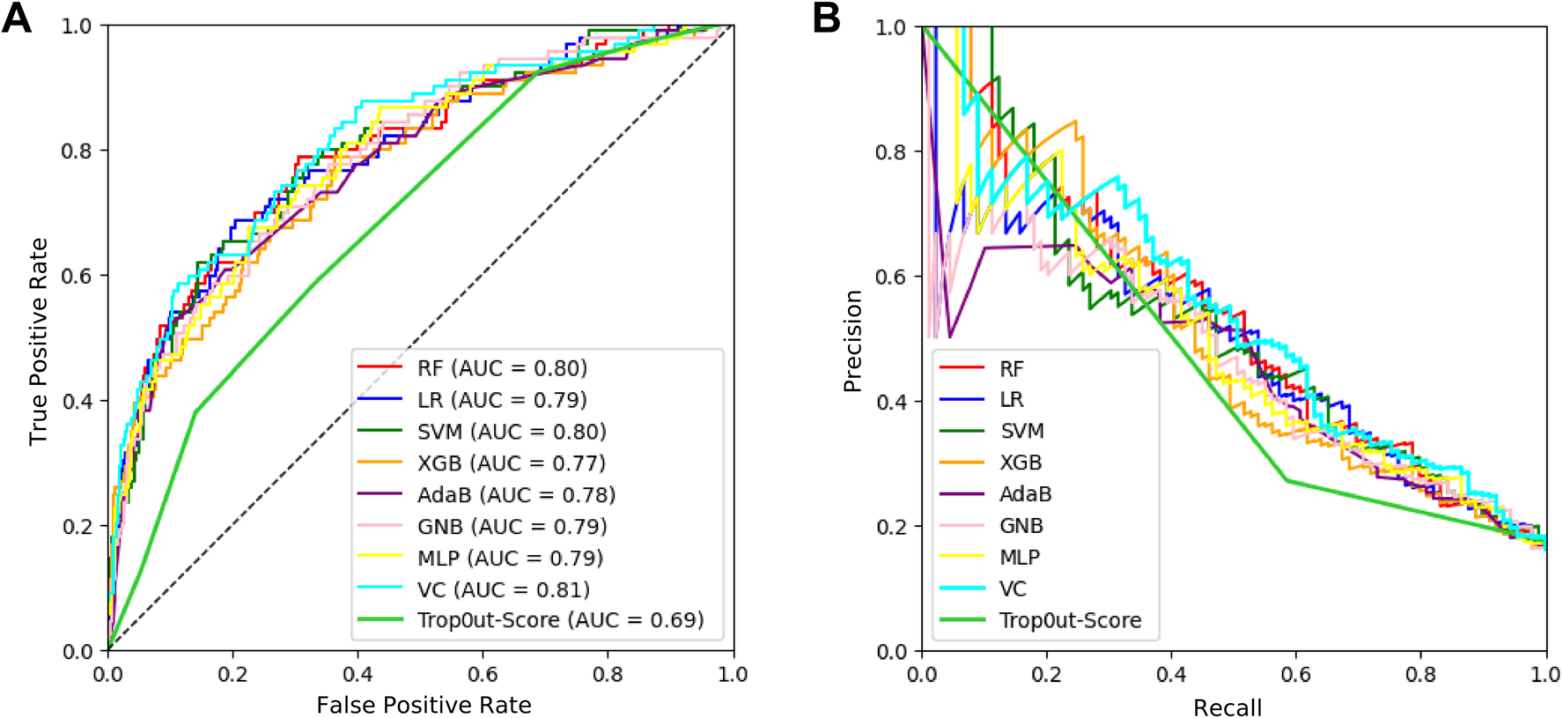
Performance comparison for 7 ML models, a voting classifier integrating all models and the Trop-out-Score for predicting ACAO. (**A**) ROC curves visualizing the false positive rate (1-specificity) on the x-axis and the true positive rate (sensitivity) on the y-axis. (**B**) Precision/Recall curves for the same models for predicting ACAO. Recall is plotted on the x-axis, precision is plotted on the y-axis. LR, logistic regression; RF, random forest; MLP, multilayer perceptron; GNB, gaussian naive bayes; SVM, support vector machine; VC, voting classifier; XGB, XGBoost; AdaB, AdaBoost.

ROC and PR curves for predicting ACAO in the validation set of ML models and Trop-out score are demonstrated in Fig. 4.

Here, the VC demonstrated the best discriminative performance for ACAO. The performance surpassed prediction of MACE with AUROC of 0.82. However, all models were also relatively equal in predicting ACAO (AUROC ranging from 0.99 to 0.81). The Trop-out score performed similarly to MACE with an AUROC of 0.69.

Additional performance statistics like precision, recall, specificity and F1 score for predicting ACAO are demonstrated in Table 3. All ML models except for the XGB showed significantly superior performance when compared to the Trop-out score (*p-values* ranging from < 0.001 to 0.018). We also noted very high specificity (ranging from 0.97 to 1) for all our models except for the GNB (specificity of 0.79).

**Table 3:** Performance of models for predicting ACAO.

Model	AUC (95% CI)	Recall (95% CI)	Precision (95% CI)	Specificity (95% CI)	F1-score (95% CI)	*p-Value*
Trop-out	0.69 (0.67–0.71)	0.65 (0.64–0.66)	0.78 (0.76–0.79)	0.66 (0.65–0.68)	0.71 (0.70–0.72)	–
LR	0.79 (0.73–0.88)	0.87 (0.84–0.90)	0.85 (0.81–0.90)	0.97 (0.98–0.98)	0.86 (0.82–0.90)	**0.003**
RF	0.80 (0.75–0.84)	0.86 (0.84–0.87)	0.84 (0.81–0.86)	0.98 (0.96–0.99)	0.84 (0.83–0.87)	**0.003**
XGB	0.77 (0.71–0.84)	0.86 (0.85–0.87)	0.85 (0.83–0.86)	0.97 (0.96–0.98)	0.85 (0.84–0.87)	0.060
AdaB	0.78 (0.71–0.82)	0.84 (0.81–0.88)	0.80 (0.73–0.87)	0.99 (0.99–0.10)	0.82 (0.77–0.87)	**0.012**
MLP	0.79 (0.74–0.83)	0.85 (0.83–0.87)	0.83 (0.80–0.86)	0.96 (0.94–0.98)	0.84 (0.82–0.87)	**0.001**
GNB	0.79 (0.71–0.86)	0.76 (0.71–0.81)	0.82 (0.76–0.88)	0.79 (0.74–0.84)	0.79 (0.74–0.84)	**0.018**
SVM	0.80 (0.74–0.82)	0.86 (0.83–0.88)	0.86 (0.83–0.89)	1.00 (1.00–1.00)	0.86 (0.83–0.88)	**0.005**
VC	0.81 (0.79–0.87)	0.88 (0.86–0.90)	0.86 (0.84–0.87)	0.97 (0.96–0.99)	0.87 (0.85–0.89)	** < 0.001**

Significant values are in [bold].

Performance of 8 ML models, as well as the Trop-out score for predicting ACAO in the validation dataset (n = 552). Models were compared to the Trop-out score and *p*-values were calculated using the De-Long test. Values in brackets show the 95% confidence intervals and were calculated via a bootstrap method.

LR, logistic regression; RF, random forest; MLP, multilayer perceptron; NB, naive Bayes; SVM, support vector machine; VC, voting classifier; XGB, XGBoost; AdaB, AdaBoost.

### Analysis of feature importance identifies specific clinical parameters as beneficial to discriminate the risk of MACE and the presence of ACAO

**Figure 5:**
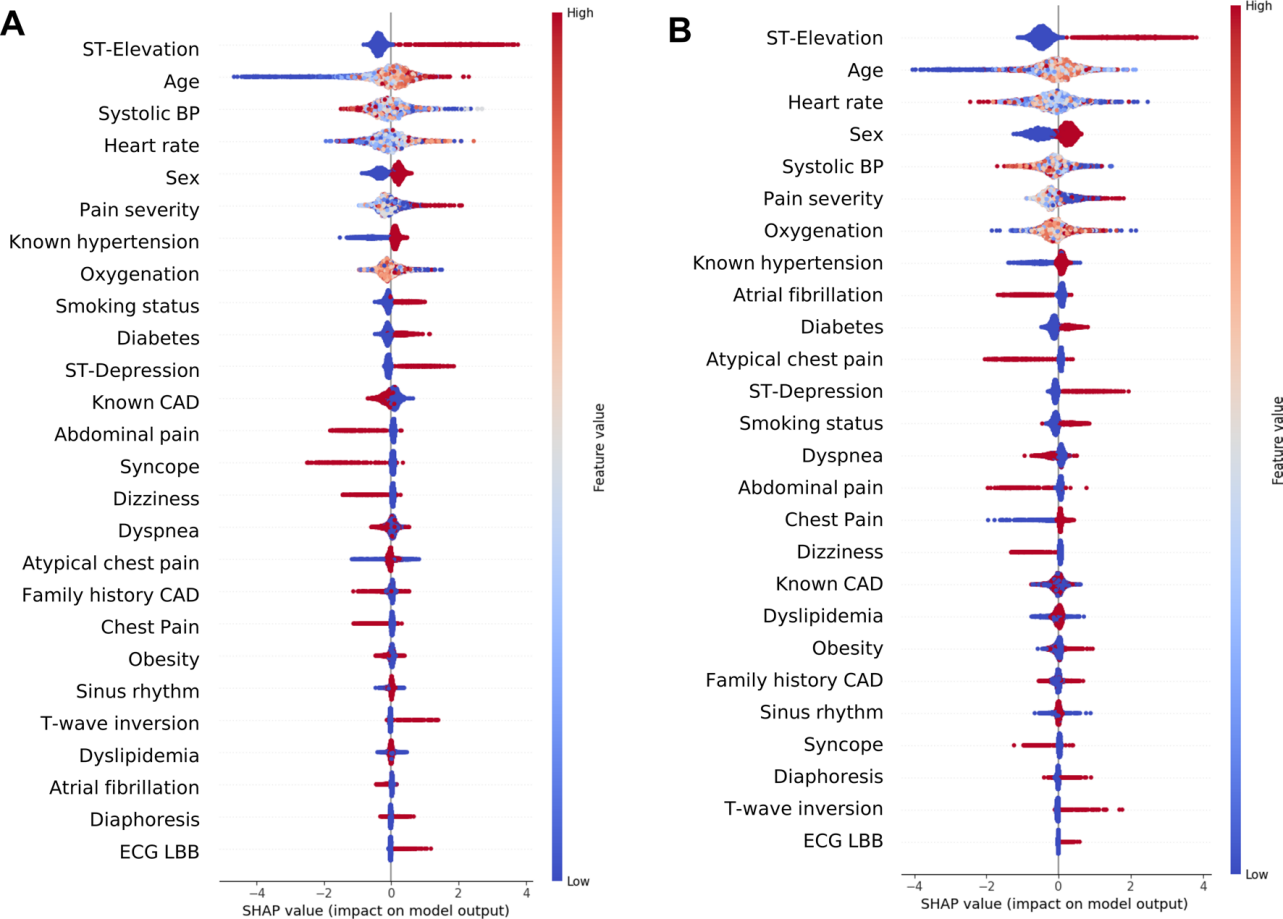
SHAP values for individual features to predict MACE (**A**) and ACAO (**B**). Feature importance is listed in descending order. Blue dots indicate low feature values, red dots indicate high feature values. Positive Shap values (right side of the middle line) indicate higher likelihood of the outcome while negative Shap values (left side of the middle line) indicate lower likelihood of the outcome. CAD, coronary artery disease; LBB, left bundle branch block; BP, blood pressure. The feature sex was coded as follows: 0 = female, 1 = male.

To increase model explainability, a SHAP analyses were carried out. SHAP values for individual features were calculated and are presented in Fig. 5A (outcome MACE) and Fig. 5B (outcome ACAO). They not only show feature importance (in descending order) but also demonstrate positive and negative relationships between feature values and outcome variable (i.e. MACE or ACAO).

Presents of ST-elevations and depressions, T-wave inversions, left bundle branch block, high pain severity and presence of risk factors like smoking and diabetes correlated with MACE whereas, young age, abdominal pain, syncope and female sex correlated with no MACE.

Unsurprisingly, presents of ST-elevations and depressions, T-wave inversions, left bundle branch block and presence of risk factors like smoking, obesity and diabetes correlated also with ACAO whereas atypical chest pain, young age, dizziness and female sex correlated with no ACAO.

## Discussion

In the present study we developed multiple machine learning models to predict major adverse cardiac events and acute coronary artery occlusion with preclinically obtained data. We then compared the performance of these models to a modified established risk score. We found that all of the ML models were superior to the “TropOut” score with the LR and the VC demonstrating the best performance in identifying MACE (AUROC 0.78). For ACAO the VC also comprised the best performance (AUROC 0.81). This is not surprising since it combines and weights the output of multiple models to optimize the predictive performance.

Quick decision-making is of utmost importance in preclinical diagnostic and treatment of patients with suspected ACS. Not every medical facility is equipped with a 24-h catheter laboratory. Therefore, a qualified assessment of early need for coronary revascularization is important in order to decide which hospital to admit the patient to and thereby guarantee optimal patient care and improve prognosis^2–4^.

Several studies have been undertaken to evaluate the predictive value of the established HEART score in an emergency setting^5,13^. Sagel et al. ^6^ even modified the score to predict MACE in a preclinical setting, thus creating the preHEART score. However, one of the HEART score components is the analysis of troponin levels. Even though the authors of the preHEART score used rapid, visual point of care immunoassays, these are unfortunately not available for emergency care providers in most areas. In order to test the performance of this score without troponin, we retrospectively calculated the “TropOut” score, a version of the preHEART score comprising medical history, ECG, age and risk factors but without troponin analysis. Unfortunately, this the TropOut score showed poor discriminatory power to identify MACE and ACAO in preclinical patients with chest pain within our study cohort.

With the use of ML algorithms, we were able to create models with vastly improved performance. As mentioned above, the VC model showed an AUROC value of 0.78 for prediction of MACE and 0.81 for ACAO. Even though this performance cannot quite hold up to the original preHEART score (AUROC = 0.85) for predicting MACE, the performance is remarkable, especially when considering that the driving key biomarker troponin was excluded in our proposed model. Since cardiac troponin has a high sensitivity for myocardial cell loss, it is very likely that it´s addition would have also significantly improved our model’s performance. Therefore, the addition of troponin essays in the preclinical setting would likely help identifying patients with ACAO or at risk for MACE even further.

We noted a significantly higher specificity compared to sensitivity for predicting both MACE and ACAO. Apparently, the model makes very reliable predictions the majority of the time but there seem to be cases which are wrongly classified as non-MACE and non-ACAO. This might be due to unspecific symptoms or atypical ECG findings which do not meet the established STEMI criteria^14,15^.

Multiple authors have used ML models for risk stratification in cardiology^9,16^. ML has been shown to identify and safely rule-out MI in an inner clinical cohort suspected of NSTEMI using multiple variables including cardiac troponin^17–19^. However, ML algorithms display limited ability to predict mortality in patients with MI^20^. To our knowledge, there have been two studies which used machine learning models to predict ACS in a purely preclinical setting. Al-Zaiti et al. tried to predict ACS only using data from a preclinical 12-lead ECG whereas Takeda et al. used vital signs, history and a 3-lead ECG to predict ACS and myocardial infarction^21,22^. Our approach is novel and different in that we chose a different secondary endpoint. MACE was chosen in order to directly compare our model to established, non-ML scores. For the preclinical management, our secondary endpoint, acute coronary artery occlusion, could be even more relevant. Myocardial infarction can be caused by different underlying pathophysiologies. Myocardial cell loss secondary to a demand–supply mismatch in oxygen not related to atherosclerotic plaque instability is known as a type II myocardial infarction^3^. However, those patients do not necessarily need immediate interventional revascularization and the broad definition of myocardial infarction therefore might be an improper endpoint. In the 2022 Expert Consensus Decision Pathway on the Evaluation and Disposition of Acute Chest Pain, the American College of Cardiology also notes that up to 40% of patients with ACAO are not correctly identified by using the STEMI criteria^14,23^. Therefore, ACAO could be a superior parameter to help decide on where to admit the patient to and whether or not to preclinically administer antiplatelet drugs. Patients with NSTEMI but especially with acute coronary artery occlusion without ST elevations on ECG have been shown to receive delayed PCI when compared to patients suffering from ST-elevation myocardial infarction and have worse outcomes^24,25^. As mentioned above, our model showed especially good predictive capabilities for ACAO.

Even though ML algorithms clearly have high potential to support decision making, our model heavily relies on medical expertise by healthcare providers. As seen in Fig. 5, the feature ST-Elevation as assessed by the emergency physician still is paramount for predicting both endpoints in our models. Not surprisingly, similar findings have been reported by Takeda et al.^21^.

SHAP analyses provides interesting insights into predictive value of symptoms, patient history and vital signs. While some features like ECG changes, age, sex and risk factor are easy to interpret, others seem more complex. In our model, diaphoresis was associated with both high and low risk for MACE and ACAO. This might be in part explained by our retrospective study design. Even though notes from the emergency protocol provide clear, dichotomous information, we cannot say if the treating physician associated the symptom diaphoresis with an ACS since the symptom can have a vastly different “Clinical Gestalt”. This could explain that our model performed worse when compared to Takeda et al. An alternative, provocative explanation could be a higher diagnostic skill level (like ECG interpretation and history taking) of paramedics when compared to physicians in a preclinical setting. Also, the patient collective could be different since the study by Takeda et al. was carried out in Japan.

Sensitivities for our model ranged from 0.70 to 0.77 for predicting MACE and 0.76–0.88 for predicting ACAO. In comparison, a meta analyzes including over 44,000 patients demonstrated a sensitivity of 0.96 for predication of MACE when a cutoff of ≥ 4 points of the heart score was used. As expected, this resulted in a rather poor specificity of 0.45%^26^.

The ideal model would demonstrate both high sensitivities and specificities. Unfortunately, in a condition like ACS and a setting were laboratory diagnostics like troponin is not available, this seems difficult to achieve. However, we have to admitted that in a life-threatening condition like ACS, false positives (i.e. poor sensitivity) are more acceptable then false negatives (i.e. poor specificity). In our models, patients were classified as positive if the predicted probability was great or equal to 0.5, and negative if otherwise. In order to enhance sensitivity, programming of our models could be adapted. Naturally, this would result in a decline in specificity. Most importantly, clinicians using tools like the one developed in our study need to be aware of the model´s strengths and limitations. As of right now, our model is not suitable for excluding ACAO or patients at risk of MACE in a preclinical collective suspected of ACS. However, it could increase emergency physician´s confidence in preclinically activating the coronary catheter laboratory for suspected ACAO.

In our district, preclinical documentation is carried out digitally with the use of tablets. Since patient history, vitals and ECG interpretation need to be inputted for documentation anyways, it would be feasible to integrate ML models. This way, the software could automatically calculate variables like sensitivities and specificities for endpoints like ACAO and MACE. Furthermore, ML has been used in ECG interpretation in a preclinical setting^22,27^. Combining those ML algorithms could potentially show a better performance and present a powerful tool in aiding preclinical health care providers on site even further.

Even in the absence of direct integration of our models into preclinical ACS diagnostics, our study has important clinical implications. Unsupervised analyses show that preclinical ACS patients are a heterogenous collective and desired endpoints are not easily identified. Even when using supervised machine learning, a high level of diagnostic skill will always be necessary since the models rely on high quality data. As mentioned before, SHAP analyses shows that out of all investigated parameters, ST-elevation is still the most important marker for properly identify ACAO and patients at risk of MACE. This highlights the necessity for a high clinical expertise and ECG interpretation skills in professionals diagnosing and treating patients with suspected ACS in a preclinical setting.

### Limitations

Our study has several limitations. For ECG interpretation, we had to rely on the emergency physician’s documentation and were not able to manually interpret the preclinical 12-lead ECG ourselves. Therefore, the quality and accuracy of this documentation might vary. Our study design relied on retrospective data collection. A predetermined questionnaire would likely improve the quality of the data and also the models’ predictive power.

Since patients could present to the emergency department on their own or in rare cases might be transferred by other providers than the cooperating rescue stations, we cannot exclude missing some cases of ACS in our study. Therefore, selection bias cannot be fully excluded.

In line with common machine learning methodology, we did validate our findings on the validation cohort. However, our algorithm has not yet been validated on external data. Especially the lack of a prospective validation cohort is the biggest limitation of our study and further analysis is needed. To our knowledge, the only comparable study which used prospectively recorded data was carried out by Takeda et al. and achieved slightly better AUROC for the endpoint ACS then our study did for MACE and ACAO (0.86 versus 0.78 and 0.81 respectively)^21^. However, because of the different preclinical emergency systems in Japan and Germany (paramedics versus emergency medicine physician), the studies are only partially comparable. Since most countries rely on paramedics for preclinical emergency medicine, our findings might not be directly transferable to other settings. At the moment, our study can only be viewed as hypothesis generating until the algorithms are prospectively validated on another patient cohort.

## Conclusion

ML algorithms using only preclinically available data and no laboratory values like troponin showed superior performance in predicting MACE until hospital discharge and ACAO compared to the TropOut score, a modified version of the preHEART score. This way, ML models could potentially help emergency medicine personnel to predict important outcomes like MACE and ACAO, facilitate immediate coronary revascularization and improve preclinical care. Furthermore, feature importance analyses demonstrated that presence of ST-elevation was the most important parameters for predicting MACE and ACAO.

### Supplementary Information

Supplementary Information.

## Data Availability

Study data is available from the corresponding author upon reasonable request.

## Abbreviations

ACS: Acute coronary syndrome
CRP: C-reactive protein
hsTnI: High sensitive troponin I
TnI: Troponin I
BMI: Body mass index
ECG: Electrocardiogram
LR: Logistic regression
RF: Random forest
VC: Voting classifier
SVM: Support vector machine
GNB: Gaussian naive bayes
XGB: Extreme gradient boosting
AdaB: Adaptive boosting
MLP: Multilayer perceptron
ß-Blockers: Beta blockers
ACE-I: Angiotensin converting enzyme inhibitor
ARB: Angiotensin receptor blocker
MRA: Mineralocorticoid receptor antagonist
ASA: Acetylsalicylic acid
OAC: Oral anticoagulation
CK: Creatinine kinase
CVRF: Cardiovascular risk factors
CHD: Coronary artery disease
DM: Diabetes mellitus
MACE: Major adverse cardiac event
ACAO: Acute coronary artery occlusion
Pro BNP: Brain natriuretic peptide
SHAP: SHapley Additive exPlanations
AUROC: Area under the receiver operating characteristics
LBB: Left bundle branch block
IQR: Interquartile range

## Code availability

All analyses were performed on publicly available software, as indicated in the “Methods” section. The code for implementing the machine learning models in Pythons is based on the vignettes provided by scikit-learn (https://scikit-learn.org/stable/supervised_learning.html#supervised-learning) with minor adaptations to our specific dataset. Detailed Python and R code can be obtained from the corresponding author upon reasonable request. Any commercial use (that includes sale, license, distribution, lease or transfer) is prohibited.
