# Supplementary material for: Preclinical identification of acute coronary syndrome without high sensitivity troponin assays using machine learning algorithms
Source: Sci Rep. 2024 Apr 29;14:9796. doi: 10.1038/s41598-024-60249-6 (PMC11058266; doi:10.1038/s41598-024-60249-6)
Supplement: Supplementary file 1 — Supplementary Information. [file 41598_2024_60249_MOESM1_ESM.docx]

# Preclinical identification of acute coronary syndrome without high sensitivity troponin assays using machine learning algorithms

Andreas Goldschmied, Manuel Sigle, Wenke Faller, Diana Heurich, Meinrad Gawaz, Karin Anne Lydia Müller^*^

Department of Cardiology and Angiology, University Hospital Tübingen, Tübingen, Germany

## *Corresponding author:

Karin Anne Lydia Mueller, MD

Department of Cardiology and Angiology

University Hospital of the Eberhard Karls University Tuebingen

Otfried-Mueller-Str.10, 72076 Tuebingen, Germany

Tel: +49-7071-29-83688

Fax: +49-7071-29-5749

E-mail: [k.mueller@med.uni-tuebingen.de](mailto:k.mueller@med.uni-tuebingen.de)

**Supplementary Table 1**

**Supplementary Figures 1-2**

**Supplementary table 1:** Available features and possible values

| **Feature** | **Nominal, ordinal or metric** | **Coding if non-metric,**  **Units if metric** |
| --- | --- | --- |
| **ST-elevation** | **Nominal** | **0= no, 1= yes** |
| **Chest pain** | **Nominal** | **0= no, 1= yes** |
| **T-wave inversion** | **Nominal** | **0= no, 1= yes** |
| **Dizziness** | **Nominal** | **0= no, 1= yes** |
| **Atypical chest pain** | **Nominal** | **0= no, 1= yes** |
| **Sex** | **Nominal** | **0= women, 1= men** |
| **Smoking status** | **Nominal** | **0= no, 1= yes** |
| **ST-depression** | **Nominal** | **0= no, 1= yes** |
| **Dyslipidemia** | **Nominal** | **0= no, 1= yes** |
| **Diaphoresis** | **Nominal** | **0= no, 1= yes** |
| **Known hypertension** | **Nominal** | **0= no, 1= yes** |
| **Abdominal pain** | **Nominal** | **0= no, 1= yes** |
| **Obesity** | **Nominal** | **0= no, 1= yes** |
| **Dyspnea** | **Nominal** | **0= no, 1= yes** |
| **Age** | **Metric** | **years** |
| **Pain severity** | **Ordinal** | **0= no pain, 10= worst pain** |
| **Diabetes** | **Nominal** | **0= no, 1= yes** |
| **Syncope** | **Nominal** | **0= no, 1= yes** |
| **Family history CAD** | **Nominal** | **0= no, 1= yes** |
| **SpO2** | **Metric** | **%** |
| **Heartrate** | **Metric** | **beats/min** |
| **Known coronary artery disease** | **Nominal** | **0= no, 1= yes** |
| **Systolic blood pressure** | **Metric** |  |
| **ECG left bundle branch block** | **nominal** | **0= no, 1= yes** |
| **Atrial fibrillation** | **Nominal** | **0= no, 1= yes** |
| **Sinus rhythm** | **Nominal** | **0= no, 1= yes** |
| Symptom onset | Metric | minutes |
| No ECG | Nominal | 0= no, 1= yes |
| ECG AV-Block type 2 | Nominal | 0= no, 1= yes |
| ECG AV-Block type 3 | Nominal | 0= no, 1= yes |
| ECG broad QRS tachycardia | Nominal | 0= no, 1= yes |
| ECG narrow QRS tachycardia | Nominal | 0= no, 1= yes |
| ECG pacemaker | Nominal | 0= no, 1= yes |
| ECG ventricular extrasystole | Nominal | 0= no, 1= yes |
| ECG other rhythm | Nominal | 0= no, 1= yes |
| ECG incomplete right bundle branch block | Nominal | 0= no, 1= yes |
| ECG right bundle branch block | Nominal | 0= no, 1= yes |
| ECG loss of R-waves | Nominal | 0= no, 1= yes |
| ECG left anterior hemiblock | Nominal | 0= no, 1= yes |
| ECG incomplete left bundle branch block | Nominal | 0= no, 1= yes |
| ECG other repolarization pattern | Nominal | 0= no, 1= yes |
| Cough | Nominal | 0= no, 1= yes |
| Emesis | Nominal | 0= no, 1= yes |
| BMI | Metric | Kg/m^2^ |

Available features and possible values. Features in bold were used in the predication of outcomes


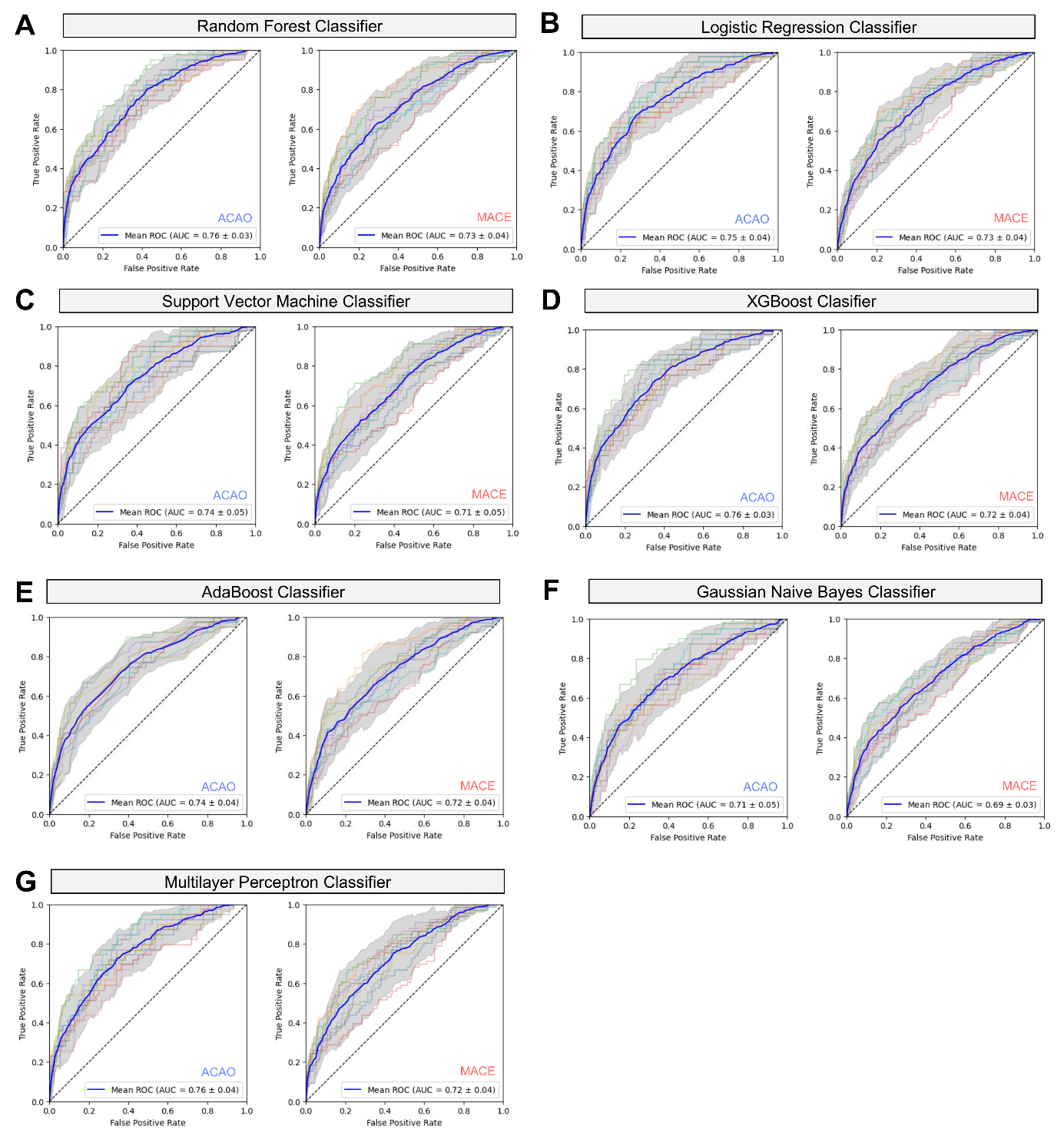


**Supplementary figure 1.** ROC curves for 10-fold cross-validation in the testing set. The blue curve represents the mean AUC. The false positive rate is plotted on the x-aces, the true positive rate is plotted on the y-axes. **(A)** RF. **(B)** LR. **(C)** SVM. **(D)** XGB. **(E)** AdaB. **(F)** GNB. **(G)** MLP. LR (logistic regression), RF (random forest), MLP (multilayer perceptron), GNB (gaussian naive bayes), SVM (support vector machine), XGB (XGBoost), AdaB (AdaBoost)


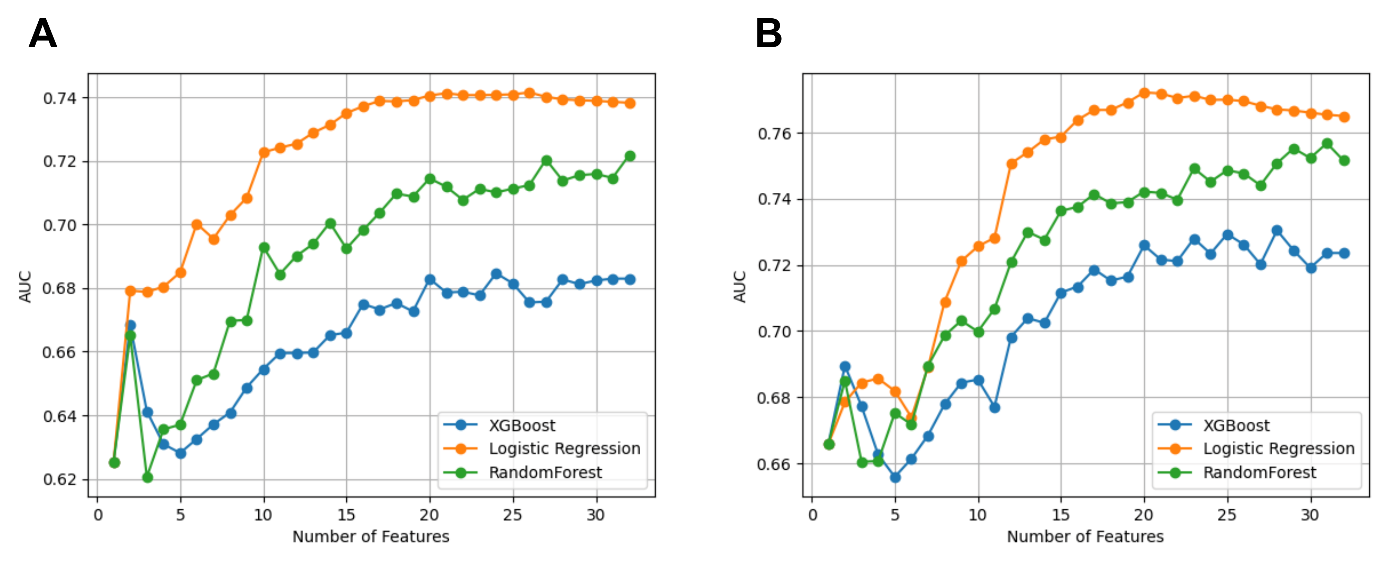


**Supplementary figure 2.** Number of features and correlation to performance of ML models for MACE **(A)** and ACAO **(B).** Numbers of features used is plotted on the x-axis, AUC for model prediction in the training set is plotted on the y-axis
